# Position Control of a Pneumatic Drive Using a Fuzzy Controller with an Analytic Activation Function

**DOI:** 10.3390/s22031004

**Published:** 2022-01-27

**Authors:** Željko Šitum, Danko Ćorić

**Affiliations:** 1 Department of Robotics and Production System Automation, Faculty of Mechanical Engineering and Naval Architecture, University of Zagreb, I. Lučića 5, 10000 Zagreb, Croatia; zeljko.situm@fsb.hr; 2 Department of Materials, Faculty of Mechanical Engineering and Naval Architecture, University of Zagreb, I. Lučića 5, 10000 Zagreb, Croatia

**Keywords:** servo pneumatic drive, fuzzy logic, activation function, position control

## Abstract

The fuzzy logic controller, which uses an analytic activation function for the defuzzification procedure, was applied to the position control of a servo pneumatic drive controlled by a proportional valve. The Gaussian shape of input fuzzy sets, with the possibility of their modification, was used to fuzzify the input signal. The control signal was determined by introducing an analytic function instead of defining the fuzzy rule base. In this way, a conventional 2-D fuzzy rule table base is modified into 1-D fuzzy defuzzification based on an analytic function to calculate the controller output. In this control algorithm, the problem of conventional fuzzy logic control, in terms of the exponential growth in rules as the number of input variables increases, is eliminated. The synthesis controller procedure is adjusted to the flow rate characteristic of the proportional valve. The developed control algorithms are verified by computer simulation and by testing on a real pneumatic rodless cylindrical drive.

## 1. Introduction

Fuzzy logic control (FLC) can be used to improve an existing classical controller solution by adding an extra layer of “intelligence” to the control strategy. The fuzzy controller activity can be adjusted to the process characteristics with modifications of the knowledge base, the shapes of fuzzy sets, the defuzzification procedure, or scaling factors [1]. Unfortunately, there is no simple and sufficiently understandable systematic procedure to predict the precise results of these modifications for each particular system, and very often, the trial-and-error method has to be used in order to obtain specific design requirements [2,3]. It is usually a difficult task to optimize fuzzy membership functions and rule base. To avoid such difficulties, some design techniques based on a self-organizing fuzzy controller [4,5] or synthesis of fuzzy and neural networks [6,7,8] have been proposed. An effective way to reduce the rule base size is to combine FLC and sliding mode control [9,10]. However, it is known that this approach has its disadvantages in selecting the right parameters of switching function and the undesired phenomenon of chattering due to high-frequency switching. In addition, since fuzzy controllers are nonlinear with more parameters, it is difficult to set the controller parameters compared to the classical controller [11]. Theoretically, with a large enough fuzzy rule base and input variables, any unknown function can be approximated, i.e., any shape of the input-to-output mapping surface and thus adopted controller action to process characteristics [12]. However, increasing the input variables will exponentially increase the fuzzy rule base. It is, therefore, necessary to find ways to cope with this inconvenient problem in the realization and implementation of the fuzzy logic controller. A large fuzzy rule base can also cause problems in computing and the practical realization of the control algorithm.

In this paper, the design and implementation of a FLC without a rule base for the position control of a servo pneumatic drive is presented. The controller output in each mapping process is determined by introducing an analytic activation function (AAF) instead of defining the fuzzy rule base [13]. In contrast to the common Mamdani-type min-max composition operator, all grades of input variables membership in all universe of discourses are calculated using a sum of products formula. The output fuzzy sets can be assumed as moving singletons with positions determined by an activation function in the fuzzification inference process. In this way, the classical defuzzification step is omitted and a direct input-to-output mapping process is possible, which can be easily implemented in the control algorithm.

Apart from this introduction, the paper is organized in the following manner. In Section 2, the experimental setup of a pneumatic servo drive controlled by a proportional valve is described. Then, the design of an FLC system employing a new analytic algorithm as a defuzzifier is presented. After that, the proposed control method is adjusted to the flow rate characteristic of the proportional control valve. Section 3 presents simulation and experimental results obtained by applying the proposed controller. The results were analyzed in Section 4. Finally, in Section 5, conclusions and comments are given.

## 2. Materials and Methods

**Figure 1:**
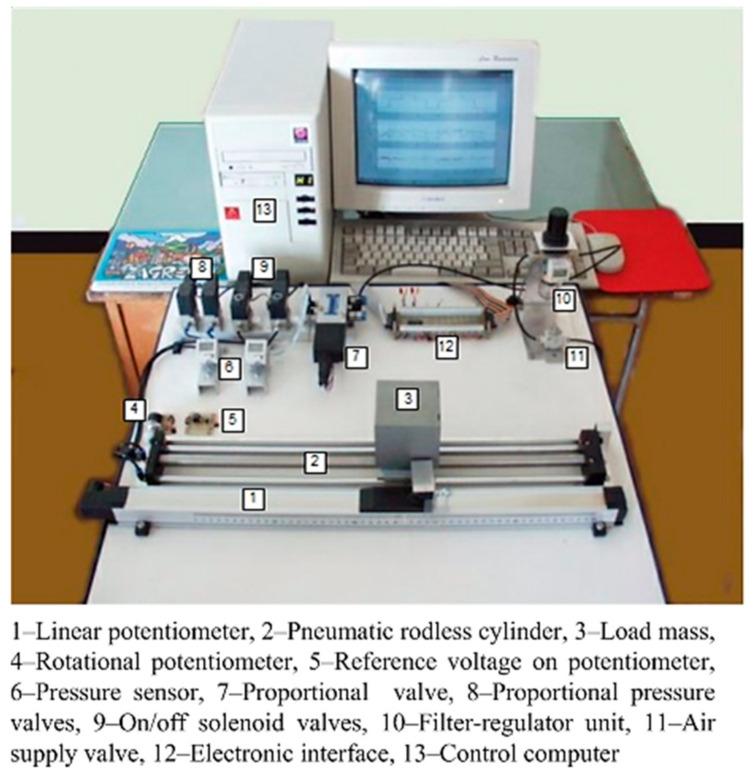
Photo of the experimental equipment.

**Figure 2:**
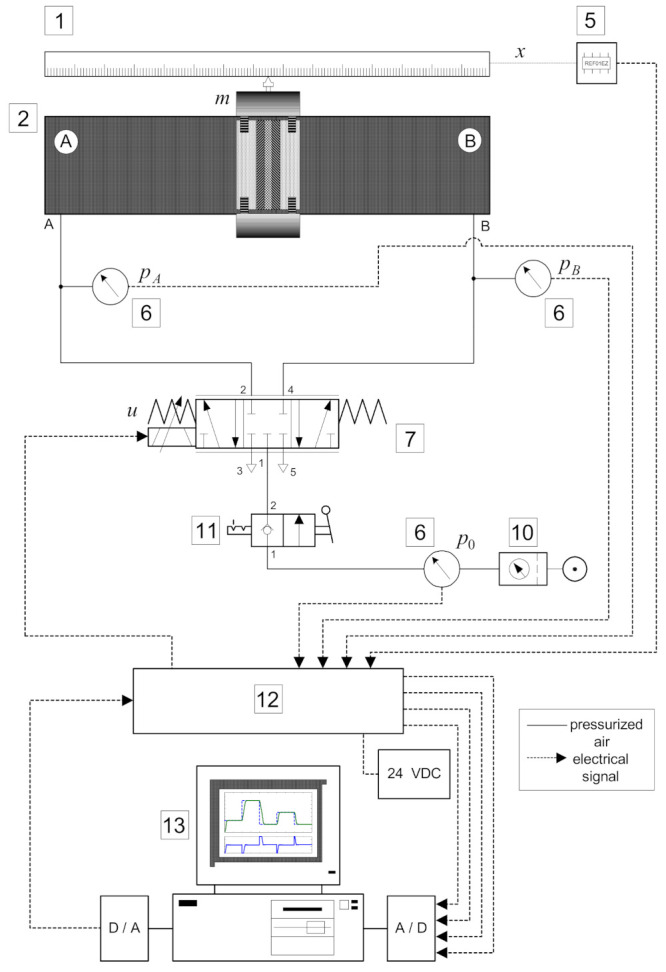
Schematic diagram of the control system.

In Figure 1, a photo of the laboratory equipment is shown, while the schematic description of the control system is illustrated in Figure 2. The experimental setup consists of standard industrial components and manually made parts. The actuator is a rodless cylinder (*SMC* CDY1S15H-500) with a stroke length of *l* = 500 mm and a diameter *d* = 15 mm. The piston position is measured by the horizontal linear potentiometer (*Festo* MLO-POT-500-TLF), which is attached to the actuator. The directly actuated proportional control valve (*Festo* MPYE-5 1/8 HF-010B), which is connected to both cylinder chambers, controls the linear motion of the piston. Three pressure transducers (*SMC* ISE4-01-26) are added to measure cylinder pressures and the pressure of the air supply.

The control software is written in C programming language and the feedback control algorithms are implemented on a Pentium-based PC using a PCL-812PG data acquisition card. All signals from the process are received in a microcomputer via a 12-bit A/D converter. The calculated control signals from the microcomputer are sent via a 12-bit D/A converter to the proportional control valve.

The experimental equipment also includes two proportional pressure valves (*SMC* VY1A00-M5) and two on/off solenoid valves (*SMC* EVT307-5D0-01F). In this paper, the control of a pneumatic drive using these valves is not considered, although research with them has been done [14,15].

### 2.1. Design of a Fuzzy Logic Controller with an Analytic Activation Function

It is well known that the conventional procedure of an FLC system design is composed of a fuzzification process, fuzzy rule base with fuzzy inference engine, and defuzzification process. In this section, a design procedure of an FLC system will be shown without any fuzzy rule base by introducing an AAF for the determination of the controller output. The proposed method is based on the theory of an adaptive FLC synthesis applied to a pneumatic servo system. In the fuzzification process, a Gaussian shape of input fuzzy sets is introduced, with the possibility of fuzzy sets modification, by employing an adjustable parameter *β*. This makes possible the setting of the distribution of input fuzzy sets, and indirectly, by using an analytic activation function, determination of the controller output in each mapping process. In this way, the analytic functions are used as a defuzzifier for generating a crisp value of controller output in each sampling time.

### 2.2. Fuzzification Process

#### 2.2.1. Definition of Fuzzy Membership Functions

For the realization of a new fuzzification process of the input variables of a fuzzy controller, it is necessary to define the fuzzy membership functions on a universe of discourse that has a modification shape possibility. In that sense, a Gaussian shape membership function of fuzzy sets with zero center positions is chosen:

(1) $$\mu (x)=K\;e^{-a\;x^{2}}$$

where *x* is the input variable of fuzzy controller and *a* is the parameter, which determines the width of the membership function $\mu \left(x\right)$. The pick value of curves is defined by the *K* coefficient. In fuzzy control theory, it is usually assumed that $\mu \left(x\right)=1$ if an input variable totally belongs to a fuzzy set $\mu \left(x=x_{c}=0\right)$, where index “c” means “center”, because of that, the coefficient *K* is set at the value *K =* 1. For different values of parameter *a*, also a different width of membership functions can be achieved. A larger parameter *a* gives a more pointed curve shape. The membership function of the input fuzzy set has the following characteristic values:

(2) $$\mu (x)=\left\{\begin{array}{ccc} 0 & \mathrm{for} & x\to -\infty \\ 1 & \mathrm{for} & x=0\\ 0 & \mathrm{for} & x\to \infty \end{array}\right.$$

Some other shapes of fuzzy sets can also be used. For example, bell-shaped, symmetric sigmoidal shaped, or cosine-shaped membership can be applied in adaptive FLC implementation. The shape of membership functions is generally less important than the number of fuzzy sets and their positions [1].

#### 2.2.2. Input Variable Normalization

The universe of discourse of the fuzzy logic controller must be large enough to cover all possible values in the control process. Therefore, in the controller design procedure, the input variable $x_{j}$ can be normalized to the interval [−1, 1] by using the following Equation:

(3) $$x_{j}^{N}=K_{j}\;x_{j\;\;\;},K_{j}=1/|x_{j\;\max}|$$

where $x_{j}^{N}$ is the normalized value of input variable $x_{j}$, j=1,…,m, and $x_{j\;\max}$ is the maximum value of $x_{j}$ on the universe of discourse. By changing the scaling factors *K_j_*, the proposed controller structure can be used in different control processes.

#### 2.2.3. Distribution of Input Fuzzy Sets

For the realization of a better adjustment possibility of input fuzzy sets, a modification of fuzzy set shape from Equation (1) is carried out by introducing the adjustable parameter *β*. Thus the grade of membership function of a normalized input variable is defined by the Equation:

(4) $$\begin{array}{l} \mu_{i}^{N}(x_{j}^{N})=\mu_{i}(x_{j}^{N})/e^{\beta \;|x_{j}^{N}|}\;\;,\\ \mu_{i}^{N}(x_{j}^{N})=K\;e^{-a\;{\left(x_{j}^{N}\right)}^{2}}/e^{\beta \;|x_{j}^{N}|}\;,\;\;\;\;\;\\ \mathrm{for}\;\;\;i=1,\ldots ,n_{j}\;,\;\;j=1,\ldots ,\;m\;. \end{array}$$

In this distribution, all input fuzzy sets have the same center positions $x_{ci}=0$ and the grade of membership function of center position is $\mu_{i}(x_{ci})=1$. In this way, the possibility of a different distribution of fuzzy sets is obtained: by modifying parameter *a* and keeping parameter *β* constant, or conversely, keeping parameter *a* constant and modifying parameter *β*, or by modification of both parameters simultaneously. This possibility of setting the shape and distribution of fuzzy sets is important for the inference process in which the activation of an output fuzzy set is determined, instead of using classical min-max operators.

### 2.3. Fuzzy Inference Engine

An input variable $x_{j}^{N}$ in the input fuzzy set *A_i_* defined on a universe of discourse has the grade of membership $\mu_{i}^{N}(x_{j}^{N})$, and activates the corresponding output fuzzy set *B_j_* with a certain degree $\mu_{B_{j}}$. As distinguished from conventional fuzzy logic controller design, the fuzzy rule-base approach and the distribution of output fuzzy sets are replaced by an analytic procedure, which calculates the positions of output fuzzy sets in the form of moving singletons. For the determination of the activation function in the inference process a *sum-prod* composition operator is used:

(5) $$\begin{array}{l} \mu_{1}^{N}\;(x_{j}^{N})\;\;\mu_{B_{j}}+\mu_{2}^{N}\;(x_{j}^{N})\;\;\mu_{B_{j}}+\;\ldots \;+\;\;\mu_{n}^{N}\;(x_{j}^{N})\;\;\mu_{B_{j}}=\\ =\sum_{i=1}^{n_{j}}\;\mu_{i}^{N}(x_{j}^{N})\;\mu_{B_{j}}=\;\;s_{j}\;\mu_{B_{j}} \end{array}$$

where *i* is the number of fuzzy sets, *j* is the number of input variables, *n_j_* is the number of membership functions of the *j*-th input variable and $\mu_{B_{j}}$ is the membership function of an output fuzzy set *B_j_*. Consequently, the activation function *s_j_* is defined by the analytic form:

(6) $$s_{j}=\sum_{i=1}^{n_{j}}\;\mu_{i}^{N}(x_{j}^{N})$$

The activation function *s_j_* indicates the grade of membership of input variable *x_j_* in all of the input fuzzy sets *A_i_*. The function for analytic determination of the controller output defined as $y_{cj}^{N}=f(x_{j}^{N})$ is then introduced instead of defining fuzzy rules, as in conventional fuzzy controller design. For the solution to this problem the following intuitive consideration is proposed: if the membership of input variable $\mu_{i}^{N}(x_{j}^{N})$ is smaller, then the distance $x_{j}^{N}$ to zero is larger. Assuming the position error *e* is the input variable, this means that the control error is larger and the control system is far from a reference position. This implies that the amplitude of the control variable should be larger. Following the same analogy, the absolute position of the corresponding output fuzzy set, which represents the controller action, should be larger. In accordance with this, amplitudes of normalized positions of output fuzzy sets can be computed by the equation:

(7) $$|y_{cj}^{N}|=1-\frac{\sum_{i=1}^{n_{j}}\mu_{i}^{N}(x_{j}^{N})}{n_{j}}=1-\frac{s_{j}}{n_{j}}$$

Because the sign of $y_{cj}^{N}$ must be equal to the sign of $x_{j}^{N}$, normalized positions of output fuzzy sets can be calculated by the expression:

(8) $$y_{cj}^{N}=(1-s_{j}/n_{j})\;\;\mathrm{sgn}(x_{j}^{N})$$

where

$$\mathrm{sgn}(x_{j}^{N})=\left\{\begin{array}{ccc} -1 & \mathrm{for} & x_{j}^{N}<0\\ 0 & \mathrm{for} & x_{j}^{N}=0\\ 1 & \mathrm{for} & x_{j}^{N}>0 \end{array}\;\right.$$

Since the input variables are normalized, if it is necessary for the regular operation of control components, the position of output fuzzy sets can be adjusted to the output domain with the scaling factor *K*_c*j*_:

(9) $$y_{cj}=K_{cj}\;(1-s_{j}/n_{j})\;\;\mathrm{sgn}(x_{j}^{N})$$

In general, the scaling factor *K*_c*j*_ of the output fuzzy set position is equal to the value of the control variable *u* for maximal control effort.

### 2.4. Defuzzification Process

To generate a non-fuzzy output (a crisp value of control signal, which is sent to the control process), a formal defuzzification procedure is used. At each sampling period, the input variable $x_{j}^{N}\left(t\right)$ activates a corresponding output fuzzy set. Such an activated output fuzzy set is in the form of a moving singleton, which represents the control signal. The controller output of multi-fuzzy input variables is the average of their individual corresponding output. The crisp value is then determined from the values of positions of output fuzzy sets obtained in Equation (9), as:

(10) $$u(t)=\frac{\sum_{j=1}^{m}y_{cj}(t)}{m}$$

The existing problem of misleading the contribution of each fuzzy input variable, in that case, demands that adjustable parameters *a* and *β* need to be designed by simulation in each concrete case. The proposed fuzzy logic controller design is very simple because a simple procedure is used for the calculation of the controller output. In contrast to a conventional rule-based approach, in this design method, the number of input variables and the number of input fuzzy sets are not limited, and the control algorithm can be realized very fast. In this way, the problem of conventional FLC systems with the exponential growth of rule base matrix by increasing the number of input variables is eliminated. For example, if we have two input variables with 10 fuzzy sets distributed to the input domain, in the conventional rule-based approach, it is necessary to make a matrix with 100 elements (rules). In the proposed method, it is necessary to calculate the grade of membership of input variables in each fuzzy set (2 variables × 10 fuzzy sets = 20 operations according to Equation (4), and two summation procedures according to Equation (6)). In the case of increasing the number of input variables, the advantage of the proposed method, in relation to the complexity of the control algorithm, becomes more important.

### 2.5. Synthesis of Adaptive FLC Using Parameter β-Adaptation Algorithm

In the case of the proposed controller synthesis, it is very important to develop a corresponding automatic procedure for the adaptation of relevant parameters of the FLC system. The parameter *β_j_* corresponds to the closed-loop gain of the *j*-th input variable of the FLC system. Increasing the *β_j_* parameter has the consequence of increasing the amplitudes of normalized positions of centers of output fuzzy sets $y_{cj}$ and vice versa. The following performance index has been introduced in order to create an optimal procedure of the parameter *β_j_*-adaptation scheme:

(11) $$I_{j}^{i}=\frac{P_{j}\;\left\|X_{j}\right\|}{\sqrt{t_{f}+1-t_{s}}}$$

where $\left\|X_{j}\right\|$ is the Euclidean norm and may be interpreted geometrically as the distance between the input variables and the origin, *P_j_* is a corresponding scaling factor, *t*_s_ is the adaptation starting time point, and *t*_f_ is the final time. Let the desired value of the performance index Equation (11) be known and be denoted as *I_dj_*. Then the adaptation algorithm for optimal parameter *β_j_*-adaptation is given by the following procedure:

(12) $$\begin{array}{l} \mathrm{IF}:\;\;I_{j}^{i}>I_{dj\;}\\ \mathrm{THEN}:\;\;\beta_{j}^{i+1}=\beta_{j}^{i}+\delta_{j}I_{j}^{i}\;\\ \mathrm{ELSE}\;\mathrm{IF}:\;\;I_{j}^{i}<\phi_{j}\;I_{dj\;}\\ \mathrm{THEN}:\;\beta_{j}^{i+1}=\;\beta_{j}^{i}-\delta_{j}I_{j}^{i}\;\\ \mathrm{ELSE}:\;\;\beta_{j}^{}=\;\beta_{j}^{i+1}=\beta_{j}^{i}\;\; \end{array}$$

where $\delta_{j}$ is an adaptation gain for the determination of an adaptation speed (typical range $0.1<\delta_{j}>0.1$), and $\varphi_{j}$ is a parameter for the determination of a free region of the index of performance $I_{j}^{i}$ (typical range $0.5<\varphi_{j}>1$). This algorithm is the gain adaptation function, which depends on the state errors and previous control variables, and acts as a system-stabilizing factor.

### 2.6. The Adjustment of the Controller Action to the Valve Flow Rate Characteristic

**Figure 3:**
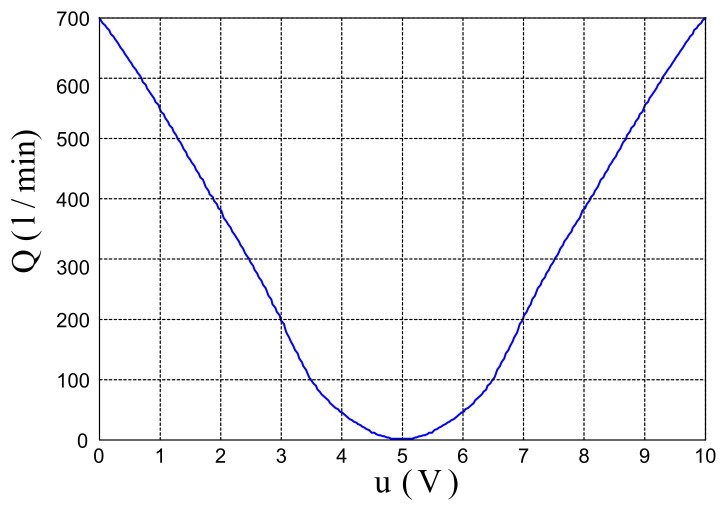
The characteristic curve for the valve flow rate.

The control signal as an output of the fuzzy controller is sent via a D/A converter to the proportional valve, which is the control component in the system. Control voltage on the valve should be in the range of 0–10 V, and a neutral position of the valve (where fluid flow to the cylinder chambers is blocked) is defined by a 5 V control signal, Figure 3.

The control signal should be adjusted to the valve flow rate characteristic so that the valve has a value of 5 V at the operating point, when the cylinder is in the reference position and control error vanishes. From Equation (6), it is noticeable that the activation function is obtained by a summation process of grades of membership of input variables to fuzzy sets. Taking this into account, we proposed the input domain distribution to 5 fuzzy sets with the center positions of membership functions is zero.

At the operating point, the activation function from Equation (6) will have a sum equal to 5, and in the realization of the control algorithm, it will correspond to a 5 V control signal. The activation function for determination of the membership degree of the input variable *x_j_* to all fuzzy sets is then given as:

(13) $$s_{j}=\sum_{i=1}^{n_{j}}\mu_{i}(x_{j})$$

where *j* = 1,…, *m* is the number of input variables, and *n_j_* = 5 is the total number of fuzzy sets on the input domain. The control signal obtained on the basis of normalized absolute values of positions of output fuzzy sets in Equation (9) is applicable for control components with a neutral position at zero (for example, DC servo motor). The proportional valve used in this work has a neutral position for 5 V control signal. Thus, the control signal should be adjusted to the valve characteristic. Taking into account the valve flow rate characteristic from Figure 3, the expression (9) is modified in order to obtain a negative slope of control function, with shifted neutral position. The modified expression of Equation (9) is then:

(14) $$y_{cj}=K_{cj}\;(\frac{s_{j}}{n_{j}}-1)\;\;\mathrm{sgn}(x_{j})\;\;+\;\;Y_{0}$$

where *Y*_0_ is the shifted neutral position from zero. The verification of the numerical values for the position of output fuzzy sets for three characteristic values of the input variable can be performed according to the following consideration. The voltage from the linear potentiometer can be in the range 0–10 V, and accordingly, the control error *e*, as an input variable $x_{j}$, can have a maximal value of +10 V or −10 V. By inserting the scaling factor, the total number of membership functions and shifted neutral position on value 5 (i.e., $K_{cj}=5$, $n_{j}=5$, $Y_{0}=5$) then from Equation (14), for three characteristic points of position of output fuzzy set *y_cj_*, we have the following values:

(15) $$\left\{\begin{array}{l} e=10,\;\;s_{j}=0\;,\;\;x_{j}>0,\;\;\mathrm{sgn}(x_{j})=1\;\;\;\;\;\;\;\;\;\;\;\to \;y_{cj}=0\;\;\;\;(\max.\;\;\mathrm{flow}\;\mathrm{to}\;\mathrm{the}\;\;\mathrm{chamber}\;A)\;\\ e=0,\;\;s_{j}=5\;,\;\;x_{j}=0,\;\;\mathrm{sgn}(x_{j})=0\;\;\;\;\;\;\;\;\;\;\;\;\;\to \;y_{cj}=5\;\;\;\;(\mathrm{blocked}\;\;\mathrm{flow})\\ e=-10,\;\;s_{j}=0\;,\;\;x_{j}<0,\;\;\mathrm{sgn}(x_{j})=-1\;\;\;\;\to \;y_{cj}=10\;\;\;\;(\max.\;\;\mathrm{flow}\;\mathrm{to}\;\mathrm{the}\;\;\mathrm{chamber}\;B) \end{array}\right.$$

This means that if the control error has a maximal value, the system is far from a reference value, and according to Figure 3, the airflow through the valve should also be at the maximal value. When we have more than one input variable, the values for the activation function *s_j_* and the values for the number of membership functions *n_j_* should be multiplied by the number of input variables. In that case, the same values for the characteristic points of output fuzzy set position *y_cj_* will be obtained. Between these characteristic points, the shape of the control function depends on the distribution of membership functions to fuzzy sets on the input domain. The position of output fuzzy sets will determine the value of the fuzzy controller output, which is sent to the proportional valve.

## 3. Results

**Figure 4:**
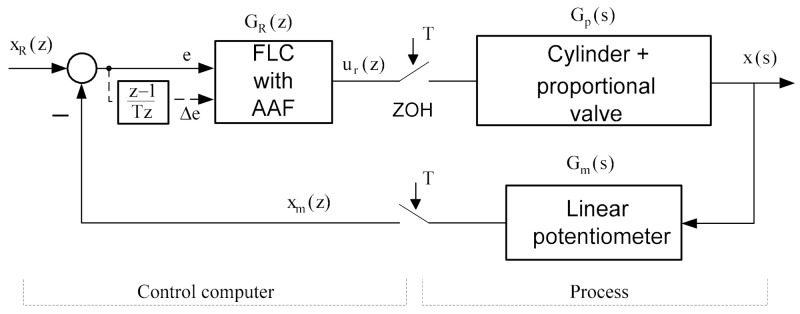
Block diagram representation of the pneumatic servo system.

The real discrete-time control system that uses fuzzy logic controller with an analytic activation function can be shown in Figure 4.

A simplified dynamic model, which still reflects the essential characteristics of a pneumatic cylinder controlled by a proportional valve, can be presented by third-order transfer function with forward gain $C_{0}$, natural frequency $\omega_{0}$ and damping ratio *ζ* as the characteristics of the system, as follows:

(16) $$G_{p}\left(s\right)=\frac{C_{0}\;\omega_{0}^{2}}{s\;\left(s^{2}+2\;\zeta \;\omega_{0}\;s+\omega_{0}^{2}\right)}$$

Delay from the D/A conversion is expressed using first-order transfer function, where *K*_DA_ is the gain of the D/A converter and *T* is the sampling time:

(17) $$G_{\mathrm{DA}}\left(s\right)=\frac{K_{\mathrm{DA}}}{\left(T/2\right)\;s+1}$$

Cylinder positions are converted to digital data using A/D converter gain *K*_AD_. Then:

(18) $$G_{\mathrm{AD}}\left(s\right)=K_{\mathrm{AD}}$$

The dynamic of the measuring system is approximated as a proportional gain *K*_m_. This model was used for the computer simulation analysis. The parameters of the control system were as follows: $\omega_{0}$ = 32.97 rad/s, *ζ* = 1.1, $C_{0}$ = 0.7 m/Vs, *K*_m_ = 20 V/m, *K*_DA_ = 2.44 × 10^−3^ V, *K*_AD_ = 204.8 V^−1^, *T* = 0.01 s and these parameters have been derived in [14].

**Figure 5:**
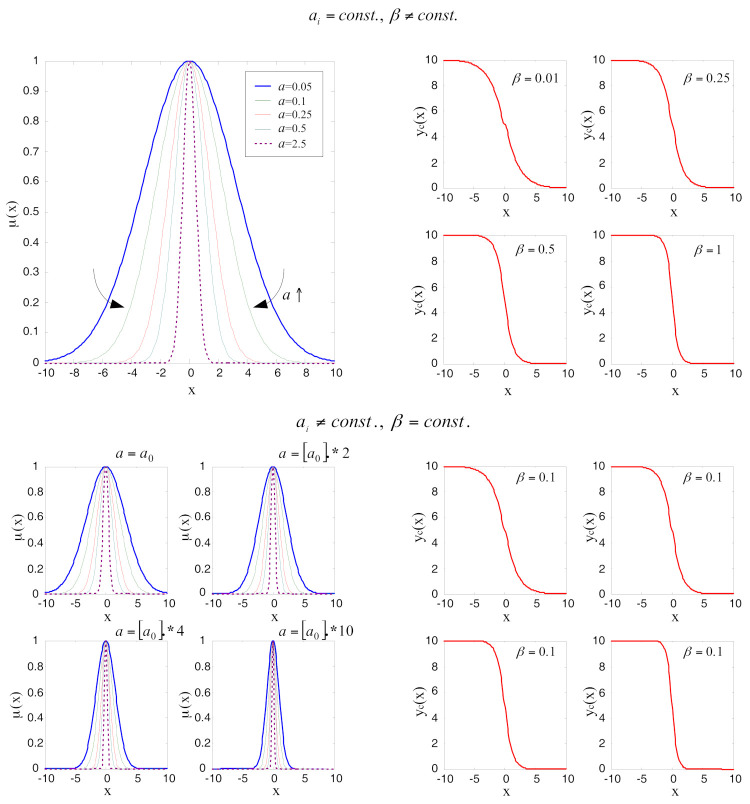
The graphic presentation of different distributions of input fuzzy sets and output control function for different off-line setting *a* and *β* parameters.

To obtain better closed-loop responses, the function of the nonlinear mapping of input variable *x_j_*(*t*) to output variable *u*(*t*) should be adjusted in the control process. The slope and amplitude of the control function can be modified by changing the distribution of membership functions of input fuzzy sets (parameter *a*) or by changing the width of membership functions (parameter *β*), as shown in Figure 5.

Increasing of *a* and *β* parameters has an influence on the narrowing of membership functions of fuzzy sets, and thus on increasing the slope of the control function. This corresponds to the increase of the closed-loop gain in a conventional controller design. The initial values of parameter *a* are given in vector *a*_0_ = (0.05 0.1 0.25 0.5 2.5), which is selected so that the membership functions of fuzzy sets appropriately cover the input domain. The distribution of input fuzzy sets in Figure 5 is given in the range of possible values of measured signals from the linear potentiometer. Based on the proportional valve flow characteristics, the output control function is presented in the range 0 to 10 with an inflexion point in 5, which corresponds to the neutral position of the proportional valve. In the control algorithm, these values will take a meaning of the control signal measured in Volts. The output control functions are smooth curves despite the input fuzzy sets do not cover the input domain in a strictly harmonized manner. The control signal amplitude is related to fluid flow in the cylinder chambers. Thus, to bring the closed-loop system to its reference position faster, it is reasonable to enable the maximum valve opening in case of a large control error.

**Figure 6:**
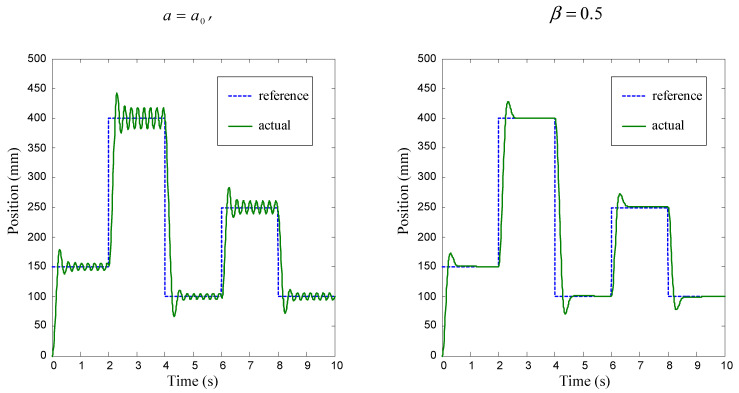

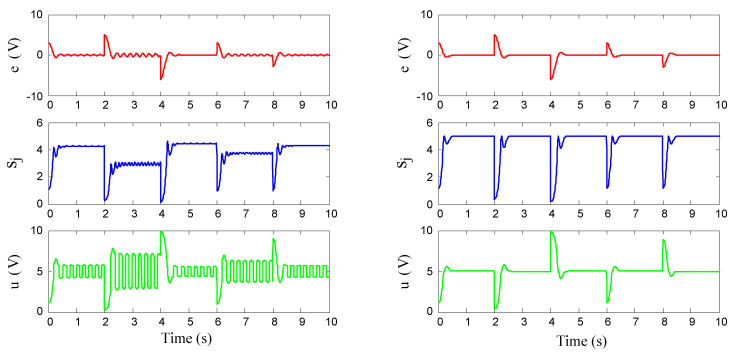
Simulation results for position control. Position error *e* is the input variable.

The simulation results of the position control of the pneumatic servo system for fixed distribution of membership functions of fuzzy sets (*a* = *a*_0_) and variable parameter *β* is shown in Figure 6. From Figure 6, it can be seen that the larger parameter *β* will cause a more oscillatory response of the system. Expanding the controller structure by the change of error Δ*e* as an additional input variable can reduce the overshoot in the system response.

This control structure, with two input variables and one output variable, corresponds to the structure of a conventional fuzzy PD controller. In this controller synthesis procedure, it is possible to set the arrangement and shape of membership functions independently by changing the parameters *a* and *β*, in order to get a better system performance.

**Figure 7:**
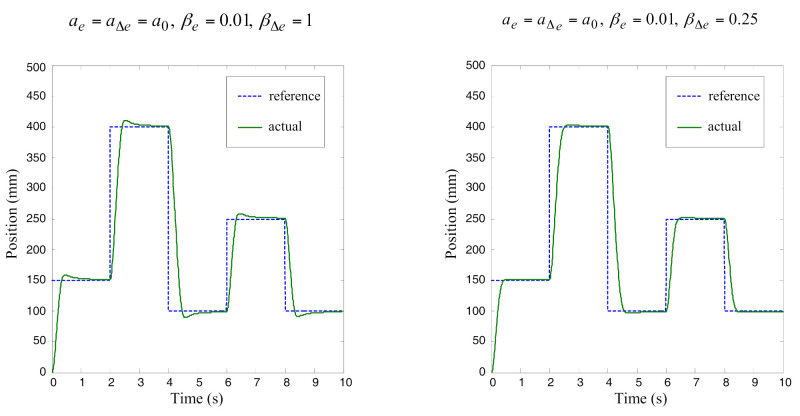

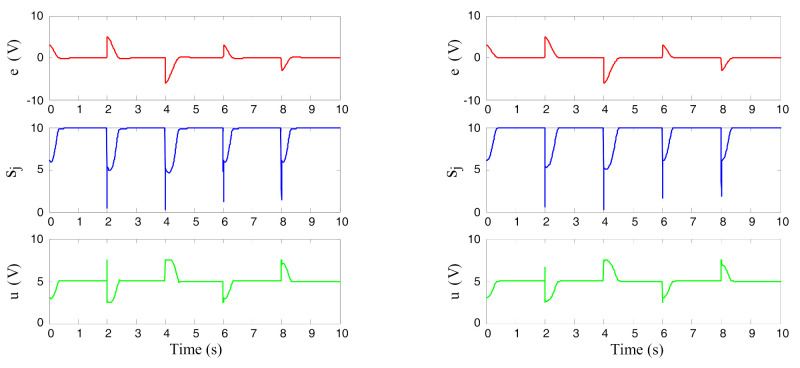
Simulation results for position control. Position error *e* and change of position error Δ*e* are the input variables.

In Figure 7, the simulation results for two cases of settings *a* and *β* parameters are shown. In both cases, parameter *a* is defined with the vector *a*_0_. The procedure for adjusting the control signal is very intuitive and does not require much iteration until a satisfactory response is achieved.

**Figure 8:**
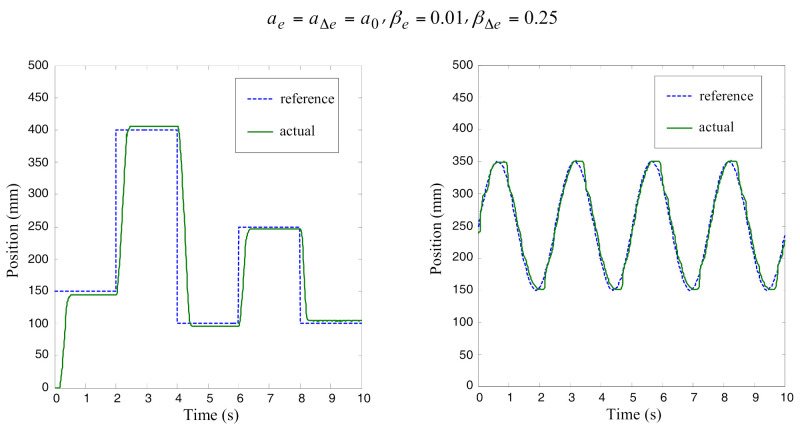

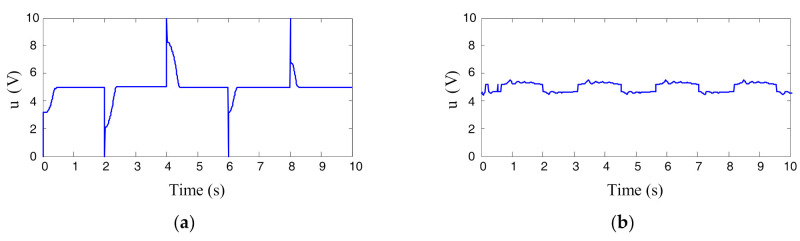
Experimental results for position control with two input variables *e* and Δ*e*: (**a**) step inputs, (**b**) sinusoidal inputs.

The control algorithms were also verified by experimental tests. The results of position control on varying step reference signals are shown in Figure 8. The experiments for tracking control were made with two input variables to the proposed fuzzy controller for a square-wave reference signal as well as for a sinusoidal reference signal. During the experiments, it turned out that the system was more sensitive to a change of the membership functions’ arrangement (parameter *a*) than to a change of shape of the membership functions (parameter *β*). The reason for this is that parameter *β* mostly modifies the control function in the narrow range of the operation point and can be used for fine-tuning the controller action.

## 4. Discussion

Generally, to get the control system more sensitive to the small range of input signals, it is reasonable to choose a wider distribution of membership functions in view of the first input variable *e*(*t*) and narrower distribution of membership functions in view of the second input variable Δ*e*(*t*); this way, the amplitude of the control signal will be smaller. This has a positive influence on reducing the system overshoot but also has a negative influence on increasing the steady-state error. Thus, a fine-tuning procedure is desirable to get the required control system performances.

## 5. Conclusions

Instead of defining a conventional fuzzy rule base, the controller output was determined by introducing an analytic activation function in the fuzzification process. The proposed controller design without the fuzzy rule base is simpler than a conventional fuzzy logic controller design. Therefore, easy implementation of the control algorithm is possible. In the proposed control method, the problem of conventional FLC in terms of the exponential growth in rules as the number of input variables increases is eliminated. The controller has been implemented to position control of a pneumatic servo drive controlled by a proportional valve. The effectiveness of the control method has been demonstrated by tracking variable reference inputs both in the simulation and the experimentation. The experimental study has shown that the controller performed well, and the system had a satisfactory output response.

## Data Availability

Data sharing is not applicable.
